# Suppression of Intestinal Epithelial Cell Chemokine Production by *Lactobacillus rhamnosus* R0011 and *Lactobacillus helveticus* R0389 Is Mediated by Secreted Bioactive Molecules

**DOI:** 10.3389/fimmu.2018.02639

**Published:** 2018-11-14

**Authors:** Michael P. Jeffrey, Janice L. Strap, Holly Jones Taggart, Julia M. Green-Johnson

**Affiliations:** ^1^Applied Bioscience Graduate Program, University of Ontario Institute of Technology, Oshawa, ON, Canada; ^2^Faculty of Science, University of Ontario Institute of Technology, Oshawa, ON, Canada; ^3^Faculty of Health Sciences, University of Ontario Institute of Technology, Oshawa, ON, Canada

**Keywords:** *Lactobacillus rhamnosus* R0011, *Lactobacillus helveticus* R0389, PRR, IL-8, CINC-1, chemokine, secretome

## Abstract

Host intestinal epithelial cells (IEC) present at the gastrointestinal interface are exposed to pathogenic and non-pathogenic bacteria and their products. Certain probiotic lactic acid bacteria (LAB) have been associated with a range of host-immune modulatory activities including down-regulation of pro-inflammatory gene expression and cytokine production by IEC, with growing evidence suggesting that these bacteria secrete bioactive molecules with immunomodulatory activity. The aim of this study was to determine whether two lactobacilli with immunomodulatory activity [*Lactobacillus rhamnosus* R0011 (Lr) and *Lactobacillus helveticus* R0389 (Lh)], produce soluble mediators able to influence IEC responses to Pattern Recognition Receptor (PRR) ligands and pro-inflammatory cytokines [Tumor Necrosis Factor α (TNFα), Interleukin-1β (IL-1β)], signals inducing IEC chemokine production during infection. To this end, the effects of cell-free supernatants (CFS) from Lr and Lh on IEC production of the pro-inflammatory chemokines interleukin (IL)-8 and cytokine-induced neutrophil chemoattractant 1 (CINC-1) induced by a range of host- or pathogen-derived pro-inflammatory stimuli were determined, and the impact on human HT-29 IEC and a primary IEC line (rat IEC-6) was compared. The Lr-CFS and Lh-CFS did not significantly modulate basal IL-8 production from HT-29 IECs or CINC-1 production from IEC-6 cells. However, both Lr-CFS and Lh-CFS significantly down-regulated IL-8 production from HT-29 IECs challenged with varied PRR ligands. Lr-CFS and Lh-CFS had differential effects on PRR-induced CINC-1 production by rat IEC-6 IECs, with no significant down-regulation of CINC-1 observed from IEC-6 IECs cultured with Lh-CFS. Further analysis of the Lr-CFS revealed down-regulation of IL-8 production induced by the pro-inflammatory cytokines IL-1β and TNFα Preliminary characterization of the bioactive constituent(s) of the Lr-CFS indicates that it is resistant to treatment with DNase, RNase, and an acidic protease, but is sensitive to alterations in pH. Taken together, these results indicate that these lactobacilli secrete bioactive molecules of low molecular weight that may modulate host innate immune activity through interactions with IEC.

## Introduction

Lactic acid bacteria (LAB) have been associated with a wide array of host-immune modulatory effects, some of which are mediated through direct interactions with host IECs. IECs express a myriad of pattern recognition receptors (PRRs) which are used to recognize bacteria and other microorganisms. PRRs include several toll-like receptors (TLR) and the cytosolic nucleotide-binding and oligomerization domain (NOD) proteins which recognize microbe-associated molecular patterns (MAMPs) produced by bacteria, fungi or viruses. Recognition of LAB by PRRs has been proposed as one mechanism through which LAB and other commensal bacteria communicate with the host (1–3). However, recent evidence suggests that the dynamic cross-talk between the host and LAB is not limited to direct interactions with host cells via PRRs and can involve soluble mediators derived from LAB [(4–6); reviewed in (7)], with many of the underlying mechanisms of action yet to be elucidated (8).

Several species of lactobacilli and bifidobacteria have been characterized for their ability to modulate immune activity and to antagonize gut pathogen attachment to IECs [reviewed in (9, 10)], and the ability of certain strains to down-regulate pro-inflammatory cytokine production by human IECs challenged with innate immune stimulants via direct contact with host cells has been characterized extensively [reviewed in (7, 11, 12)]. Some of these LAB modulate host immune activity through the inactivation of the canonical nuclear factor kappa-light-chain-enhancer of activated B cells (NF-κB) signaling pathway within IECs through direct inhibition (13) or through the up-regulation of negative regulators of NF-κB such as the A20 protein (14). Although the precise mechanisms of action behind the immunomodulatory activity of many LAB remain elusive, there has been increasing evidence to suggest that some LAB modulate activity of IECs and other cells involved in both innate and adaptive immunity through secreted molecules (15, 16). Some secreted molecules have been shown to modulate IEC cytokine production through the inactivation of NF-κB signaling by inhibiting intracellular proteasome activity (17). Other secreted proteins from certain species of lactobacilli have been attributed with cytoprotective properties. Most notably, the p75 and p40 proteins, first identified in *Lactobacillus rhamnosus* GG have been shown to protect against TNFα-induced IEC death by activating the Akt cellular pathway and through the induction of cytoprotective heat-shock proteins (18, 19). Production of amino acids and their derivatives have also been shown to be responsible for some of the effects attributed to lactobacilli. For example, lactobacilli can convert dietary tryptophan into indoles and other tryptophan metabolites, which by acting through the aryl hydrocarbon receptors present on host immune cells, can elicit a wide array of immunomodulatory activity (20, 21). Moreover, *L. reuteri* 6475 produces histamine which has been associated with inhibiting TNFα production in human monocytes via inhibition of the MAPK signaling pathway (22). Taken together, these studies emphasize the need to examine secreted products of immunomodulatory LAB to further interrogate their dynamic cross-talk with host immune cells.

In the present study, *L. rhamnosus* R0011 and *Lactobacillus helveticus* R0389 were examined for their capacity to modulate the IEC response to innate immune stimulants through the secretion of bioactive molecules, using established human and rodent IEC models. *L. rhamnosus* R0011 has been shown to antagonize gut-pathogen activity and gut-pathogen associated gastrointestinal symptoms, promote gut intestinal epithelial integrity, and improve symptoms of antibiotic-associated diarrhea in clinical trials [(23, 24); reviewed in (25)]. Transcriptomic analysis revealed that *L. rhamnosus* R0011 modulates genes involved in TLR, NOD, MAPK, and cytokine-chemokine receptor interactions in HT-29 IEC under basal conditions following exposure to live bacteria for 3 h (26). Moreover, *L. rhamnosus* R0011 has the capacity to modulate basal cytokine production from HT-29 IECs (27) and to down-regulate LPS-induced IL-8 production from HT-29 IECs *in vitro* (28). However, these studies only examined the interactions between live or dead *L. rhamnosus* R0011 with HT-29 IECs rather than the secretory activity of secreted bioactive molecules. The bacteria-free fractions of milk fermented with *L. helveticus* R0389 have been shown to have immunomodulatory activity (29, 30) and to antagonize the activity of *Salmonella enterica* serovar Typhimurium (31) suggesting that this strain produces bioactive molecules able to influence IEC activity. This study aimed to determine the role of secreted molecules produced by *L. rhamnosus* R0011 and *L. helveticus* R0389 in their interactions with IEC, by examining the impact on chemokine production induced by key PRR ligands or pro-inflammatory cytokines.

## Materials and methods

### Bacterial culture

*Lactobacillus rhamnosus* R0011 and *L. helveticus* R0389 were obtained from Lallemand Health Solutions Inc. (Montreal, QC, Canada). Lyophilized bacteria were weighed aseptically and washed 3X in sterile phosphate-buffered saline (PBS) and centrifuged at 3,000 × g for 20 min at 4°C. Following washes, the bacteria were diluted in deMan, Rogosa, and Sharpe (MRS) medium (Difco, Canada), placed in a shaking incubator at 37°C and grown to stationary phase for 17 h or until reaching an OD_620_ value of 1.60. The bacterial culture was then diluted in either non-supplemented Roswell Park Memorial Institute (RPMI)-1640 or Dulbecco's Modified Eagle's Medium (DMEM) (Sigma-Aldrich, MO, USA) medium and allowed to further propagate for an additional 23 h under the same conditions. A control consisting of MRS only, or MRS diluted in either RPMI or DMEM, was incubated concurrently with the bacterial culture. The pH of the bacterial cultures were measured (pH values of the *L. rhamnosus* R0011 and *L. helveticus* R0389 cultures were 3.90 ± 0.20 and 3.60 ± 0.10, respectively) and the pH of the acidified controls were adjusted to those of the corresponding bacterial cultures using HCl (32). Dilution to 40% v/v in RPMI-1640 for culture with HT-29 IEC or IEC-6 raised the final pH of the Lr-CFS to 5.68 ± 0.03. For preparation of the *L. rhamnosus* R0011 cell-free supernatant (Lr-CFS) and the *L. helveticus* R0389 cell-free supernatant (Lh-CFS), both the bacterial culture and the control were then centrifuged at 3,000 × g for 20 min and filtered through a 0.22 μm filter (Progene, Canada) to remove any bacteria and stored at −80°C. The filtered supernatant samples were also subjected to size fractionation using a <10 kDa Amicon Ultra−15 centrifugal filter (EMD Millipore, MA, USA). ATP concentrations in the Lr- and the Lh-CFS were measured using the ATP Determination Kit from Molecular Probes (Invitrogen, ON, Canada) and the lactic acid concentration in the Lr- and Lh-CFS was measured using the Lactic Acid Determination kit from Megazyme (Chicago, IL, USA) following manufacturer's protocols. Protein concentrations in the Lr- and Lh-CFS were determined using the Coomassie Plus Protein Assay Reagent (Thermo Fisher Scientific, MA, USA) to ensure consistency between Lr- and Lh-CFS preparations, and were 163–185 ug/mL in the undiluted CFS. A portion of the filtered secretome was plated on MRS agar to ensure that all bacteria had been removed. For some experiments, the Lr-CFS was subject to pH alteration with sodium hydroxide, boiled for 30 min, or was treated with DNase I isolated from bovine pancreas (Sigma-Aldrich, MO, USA), RNase A isolated from bovine pancreas (Sigma-Aldrich, MO, USA), or an acid-stable protease from *Rhizopus* sp. (Sigma-Aldrich, MO, USA) at concentrations of 10 μg/100 μL as described previously (4, 33). The pH of the Lr-CFS was adjusted to the optimal pH for activity of each enzyme following manufacturer's protocols. Enzymes were removed by filtration through a <10 kDa cut-off filter prior to addition to IECs. An acetone protein precipitate was also made by adding 4 volumes of chilled acetone to the Lr-CFS followed by incubation overnight at −20° C. The protein pellet was resuspended in non-supplemented RPMI-1640 medium and stored at −80°C.

### Cell culture

The HT-29 human colorectal adenocarcinoma cell line was obtained from the American Type Culture Collection (ATCC #HTB-38) and was maintained in RPMI-1640 medium supplemented with 10% fetal bovine serum (FBS) and 0.05 mg/mL gentamicin (Sigma-Aldrich, MO, USA) and were grown in 75 cm^2^ tissue culture flasks (Greiner-Bio-One, NC, USA) at 37°C, 5% CO_2_ in a humidified incubator (Thermo Fisher, MA, USA). Cells were passaged every 3 days or when cells reached ~90% confluency. Cell passages 12–28 were used for all assays.

The non-transformed rat IEC-6 (ATCC #CRL-1592) small IEC line was maintained in DMEM supplemented with 10% FBS and 0.05 mg/mL gentamicin, and cell cultures were maintained in a similar manner to HT-29 cells. Cell passages 6–15 were used for all assays.

### HT-29 and IEC-6 IEC challenge

HT-29 and IEC-6 IEC were enumerated, and viability determined using Trypan Blue following sub-culturing. Cells were then resuspended in complete culture medium and seeded at a concentration of 5 × 10^5^ cells/mL in 96-well tissue culture treated plates and placed in a CO_2_ incubator for 48 h. Cells were then exposed to defined innate immune stimulants and dilutions of the Lr-CFS, Lh-CFS, or acidified controls concurrently for 6 h at 37°C and 5% CO_2_ in a humidified incubator. Innate immune stimulants included human IL-1β (30 ng/mL) (Cedarlane, ON, Canada), human TNF-α (50 ng/mL) (PeproTech, QC, Canada), polyinosinic–polycytidylic acid sodium salt [poly (I:C)] (40 μg/mL) (Sigma-Aldrich, MO, USA), a TLR3 ligand, *S. enterica* serovar Typhimurium flagellin (100 ng/mL) (Enzo Life Sciences, ON, Canada), a TLR5 ligand, *Escherichia coli* K12 LPS (20 ng/mL) (InvivoGen), a TLR4 ligand, and L-Ala-γ-D-Glu-mDAP (Tri-DAP) (75 μg/mL) (Invivogen), a NOD1 ligand. To interrogate the mechanisms of action behind the immonodulatory activity of the Lr-CFS, HT-29 IECs were cultured with TNFα, the <10 kDa fraction of the Lr-CFS and an adenosine A_2_A receptor antagonist ((4-(2-[7-Amino-2-(2-furyl)(1,2,4)triazolo(2,3–)(1,3,5)triazin-5- ylamino]ethyl)phenol)) (ZM241385) (500 nM) (34) (Sigma-Aldrich, MO, USA), a CD73 blocker (adenosine 5′-(α,β-methylene)diphosphate) (50 μM) (34) (Sigma-Aldrich, MO, USA), or a PPARγ antagonist (GW9662) (5 μM) (35, 36) (Sigma-Aldrich, MO, USA). Following IEC challenge, supernatants were collected and stored at −80°C for cytokine quantification and IEC viabilities were determined. Cell surface molecule expression of CD73 was quantified using human anti-CD73 (Clone AD2, lot B174604, Biolegend, CA, USA) and the Millipore Guava Personal Cell Analysis (PCA) System (EMD Millipore, MA, USA).

For determination of IEC viability following challenge, the MTT cell viability/proliferation assay was used (37). Briefly, the MTT (3-(4,5-Dimethylthiazol-2-yl)-2,5-Diphenyltetrazolium Bromide) reagent (Sigma-Aldrich, MO, USA) was diluted to 0.5 mg/mL in either non-supplemented RPMI-1640 or DMEM medium. Cell culture supernatants were removed and replaced with the diluted MTT reagent. Following incubation for 3 h at 37°C, formazan crystals were dissolved using dimethyl sulfoxide and the absorbance was then measured at 600 nm. All treatments were compared to the negative control (those cells which only received cell culture medium) and the % viability, relative to the negative control, was determined.

### Cytokine quantification

IL-8 (R&D Systems, Catalog #DY208) and rat CINC-1 (R&D Systems, Catalog # DY515) were quantified from cell culture supernatants using enzyme-linked immunosorbent assays (ELISA) following manufacturer's protocols (R&D Systems, MN, USA). All ELISAs were done using 96-well high-binding Microlon 600 ELISA plates with a lower limit of quantification of 16.5 pg/mL (Greiner Bio-One, NC, USA) and plates were read at a wavelength of 450 nm using a Synergy HTTR microplate reader (Bio-Tek Instrumentation, VT, USA).

### Statistical analysis

Statistical analysis was done using GraphPad Prism's one-way analysis of variance (ANOVA) and further analysis was done using Tukey's multiple comparison test when the ANOVA indicated significant differences were present. All data are shown as the mean ± standard error of the mean (SEM) where one biological replicate (*n* = 1) is representative of three technical replicates; for most conditions, *n* = 3 or greater.

## Results

### Secreted products from *L. rhamnosus* R0011 and *L. helveticus* R0389 do not modulate constitutive IL-8 production from human HT-29 IECs or CINC-1 production from rat IEC-6 IECs

**Figure 1:**
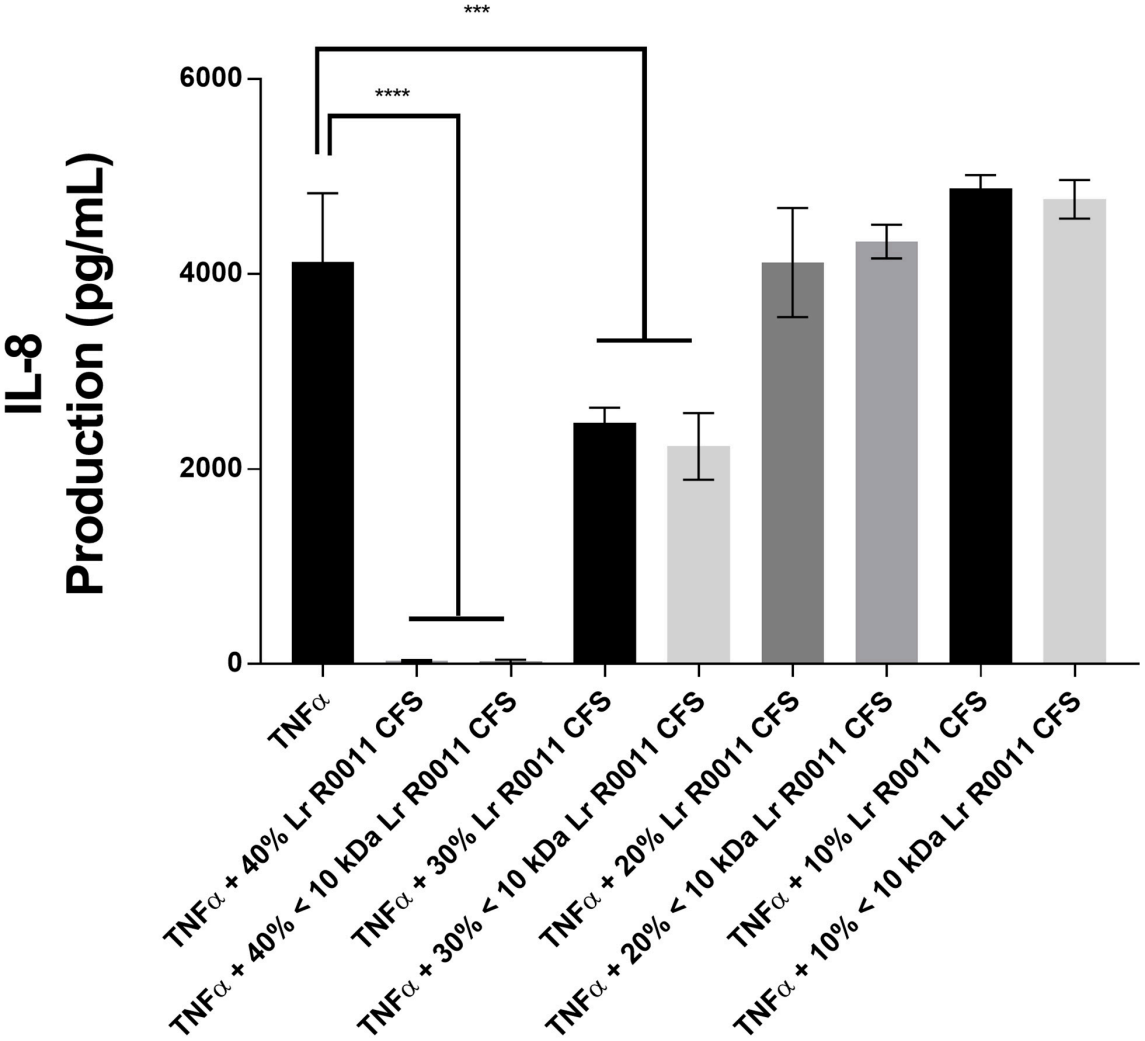
Dose-response curve for bioactivity of the *L. rhamnosus* R0011 CFS (Lr-CFS). TNFα-induced IL-8 production by HT-29 IEC cultured with different concentrations of the Lr-CFS or the <10 kDa fraction of the Lr-CFS (mean IL-8 production ± SEM) (*n* = 4). ^****^P < 0.001 and ^***^P < 0.01 as determined by the Dunnett's multiple comparisons test. Cell viability was >82% for all treatments.

**Figure 2:**
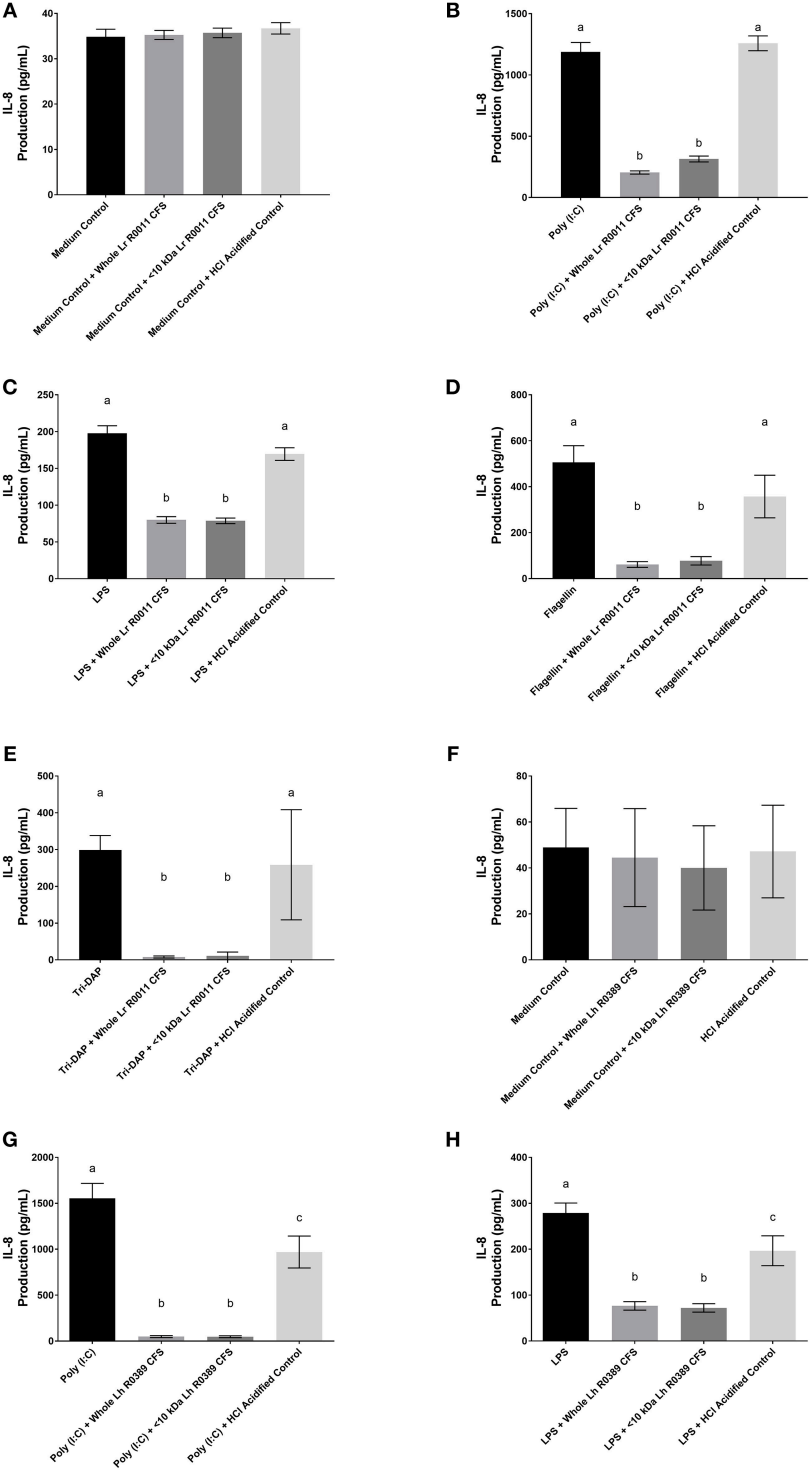
IL-8 production from human HT-29 IECs co-incubated with the Lr-CFS (*n* = 6) or the Lh-CFS (*n* = 4) and PRR ligands. Data shown is the mean IL-8 or CINC-1 production ± SEM from **(A)** HT-29 IECs cultured with the Lr-CFS, **(B)** Lr-CFS and poly(I:C), **(C)** Lr-CFS and LPS, **(D)** Lr-CFS and flagellin, **(E)** Lr-CFS and Tri-DAP, **(F)** HT-29 IECs cultured with the Lh-CFS, **(G)** Lh-CFS and poly(I:C), and **(H)** Lh-CFS and LPS. Different letters between treatments denote significance (*P* < 0.05) as determined by one-way ANOVA and Tukey's *post-hoc* test.

**Figure 3:**
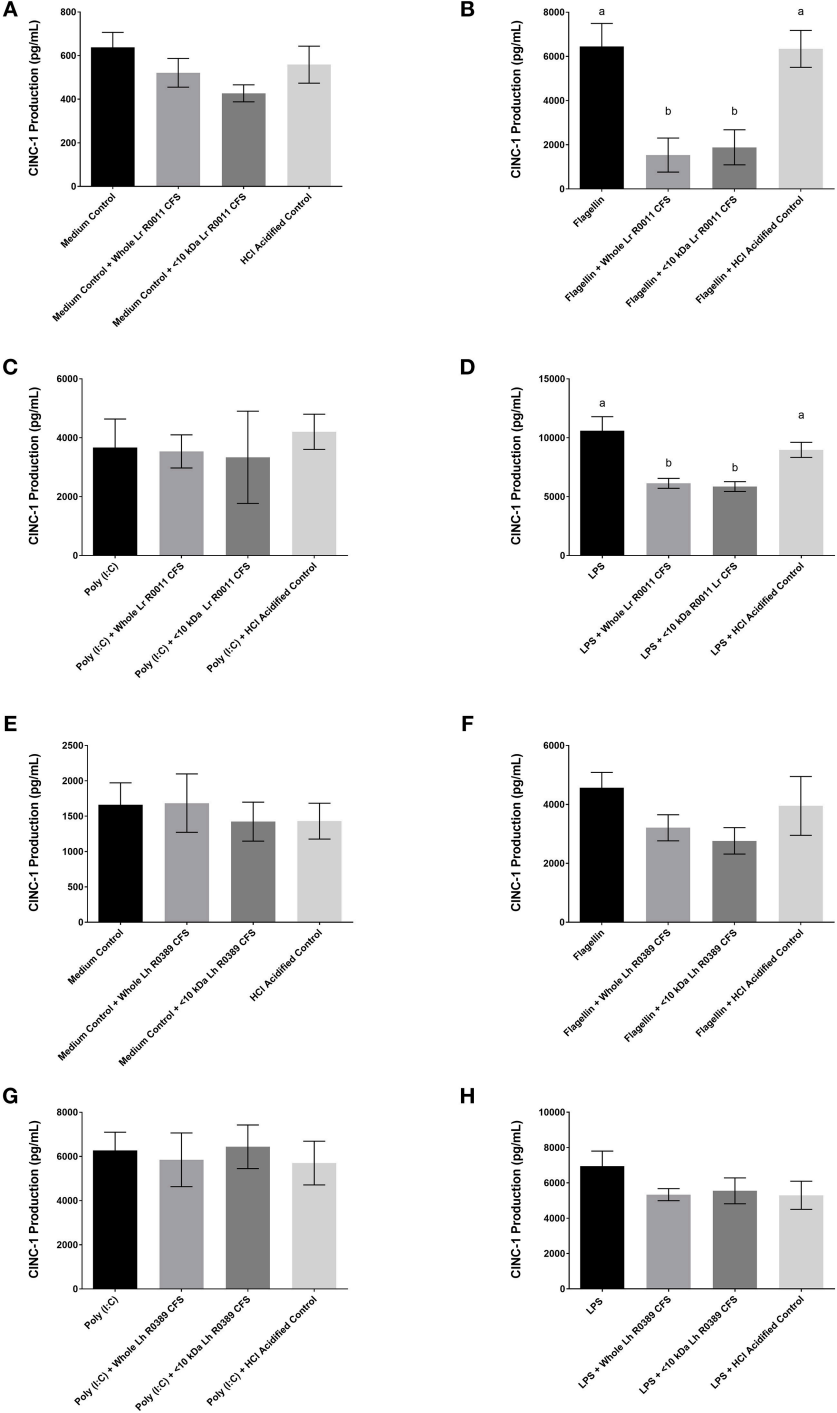
CINC-1 production from IEC-6 IECs co-incubated with the Lr-CFS (*n* = 6) or the Lh-CFS (*n* = 3) and different TLR ligands for 6 h. Data shown is the mean CINC-1 production ± SEM from IEC-6 IECs co-incubated with **(A)** IEC-6 IECs cultured with the Lr-CFS, **(B)** Lr-CFS and flagellin, **(C)** Lr-CFS and poly(I:C), **(D)** Lr-CFS and LPS, **(E)** IEC-6 IECs cultured with the Lh-CFS, **(F)** Lh-CFS and flagellin, and **(G)** Lh-CFS and poly(I:C), and **(H)** Lh-CFS and LPS. Different letters between treatments denote significance (*P* < 0.05) as determined by one-way ANOVA and Tukey's *post-hoc* test.

A dose response curve was conducted to determine the optimal concentration of the Lr-CFS to modulate IEC IL-8 production (Figure 1). A concentration of 40% v/v was selected for further analysis, based on the significant effects on IL-8 production without effects on IEC viability, which was consistently 82% or greater. In order to determine whether secreted products from *L. rhamnosus* R0011 and *L. helveticus* R0389 could increase or down-regulate constitutive IL-8 production from HT-29 IECs or CINC-1 production from IEC-6 IECs, concentrations of these chemokines were measured after IEC culture with 40% v/v of the Lr-CFS or Lh-CFS for 6 h. The Lr-CFS and the Lh-CFS did not affect constitutive IL-8 production from HT-29 IEC or constitutive CINC-1 production from IEC-6 IECs (*P* > 0.05) (Figures 2A,F, 3A,E).

### The Lr-CFS and the Lh-CFS and their <10 kDa fractions significantly decrease IL-8 production from PRR-stimulated HT-29 IECs

HT-29 human IECs were challenged with LPS, a TLR-4 ligand, poly (I:C), a TLR-3 ligand, flagellin, a TLR-5 ligand, or Tri-DAP, a NOD-1 ligand, and cultured with 40% v/v of the Lr-CFS or its <10 kDa fraction and effects on IL-8 production were measured. The Lr-CFS and its <10 kDa fraction significantly down-regulated poly (I:C)-induced IL-8 production (*P* < 0.001) (Figure 2B), LPS-induced IL-8 production (*P* < 0.001) (Figure 2C), flagellin-induced IL-8 production (*P* < 0.001) (Figure 2D), and Tri-DAP-induced IL-8 production (*P* < 0.001) (Figure 2E). This down-regulation was not observed in the presence of the acidified control, indicating that the observed down-regulatory activity of the Lr-CFS was not simply due to acidification of the extracellular environment. A similar effect was also seen in HT-29 IECs co-incubated with poly (I:C) and LPS and the Lh-CFS (Figures 2G,H). The pH-matched control for the Lh-CFS reduced PRR-induced IL-8 production from HT-29 IECs but did not down-regulate IL-8 production to the same degree as the Lh-CFS.

### The Lr-CFS and the Lh-CFS have differential effects on TLR-induced CINC-1 production from rat IEC-6 IECs

To determine the impact of the Lr-CFS and the Lh-CFS on PRR-induced CINC-1 production by primary IEC, IEC-6 cells were challenged with flagellin, poly (I:C), or LPS and incubated with the Lr-CFS or Lh-CFS or their <10 kDa fractions. The Lr-CFS and its <10 kDa fraction significantly down-regulated flagellin-induced CINC-1 production (*P* < 0.001) (Figure 3B) and LPS-induced CINC-1 production (*P* < 0.001) (Figure 3D). In contrast, the Lr-CFS or its <10 kDa fraction did not down-regulate poly (I:C)-induced CINC-1 production from challenged IEC-6 cells (*P* > 0.05) (Figure 3C), and the Lh-CFS did not significantly down-regulate CINC-1 production from IEC-6 IECs induced by any of the TLR ligands tested (Figures 3F–H), with acidification having no effect on chemokine production.

### The Lr-CFS and its <10 kDa fraction significantly down-regulates IL-1β and TNFα-induced IL-8 production from HT-29 IECs

**Figure 4:**
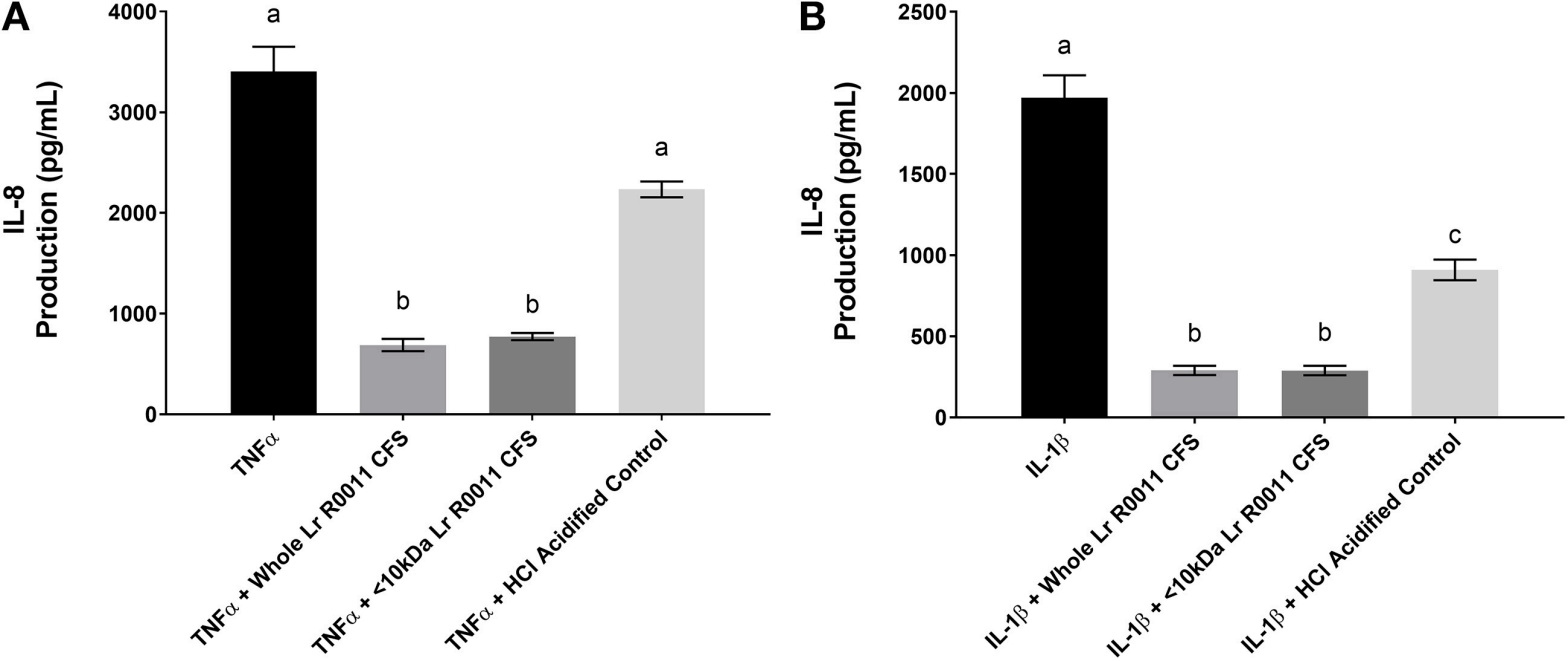
IL-8 production from HT-29 IECs co-incubated with the Lr-CFS (*n* = 6) and the pro-inflammatory cytokines TNFα or IL-1β. Data shown is the mean IL-8 production ± SEM from HT-29 IECs co-incubated with **(A)** Lr-CFS and TNF-α **(B)** Lr-CFS and IL-1β. Different letters between treatments denote significance (*P* < 0.05) as determined by one-way ANOVA and Tukey's *post-hoc* test.

Pro-inflammatory cytokine production is often an outcome of TLR-mediated signaling, and IEC are also exposed to these host-derived signals during infection. To further elucidate the effects of the Lr-CFS on the IEC response to these host-derived signals, IECs were challenged with the potent pro-inflammatory cytokines IL-1β and TNFα and cultured with Lr-CFS, or its <10 kDa fraction. TNFα-induced IL-8 production (*P* < 0.001) (Figure 4A) and IL-1β-induced IL-8 production (*P* < 0.001) (Figure 4B) from HT-29 IECs was significantly down-regulated by the Lr-CFS and its <10 kDa fraction. TNFα-induced IL-8 production was not significantly affected by acidification, and while HCl acidified controls down-regulated IL-1β-induced IL-8 production, it was not to the same extent as the Lr-CFS.

### pH neutralization of the Lr-CFS and its <10 kDa fraction abrogates its effects on TNFα-induced IL-8 production by HT-29 IECs

**Figure 5:**
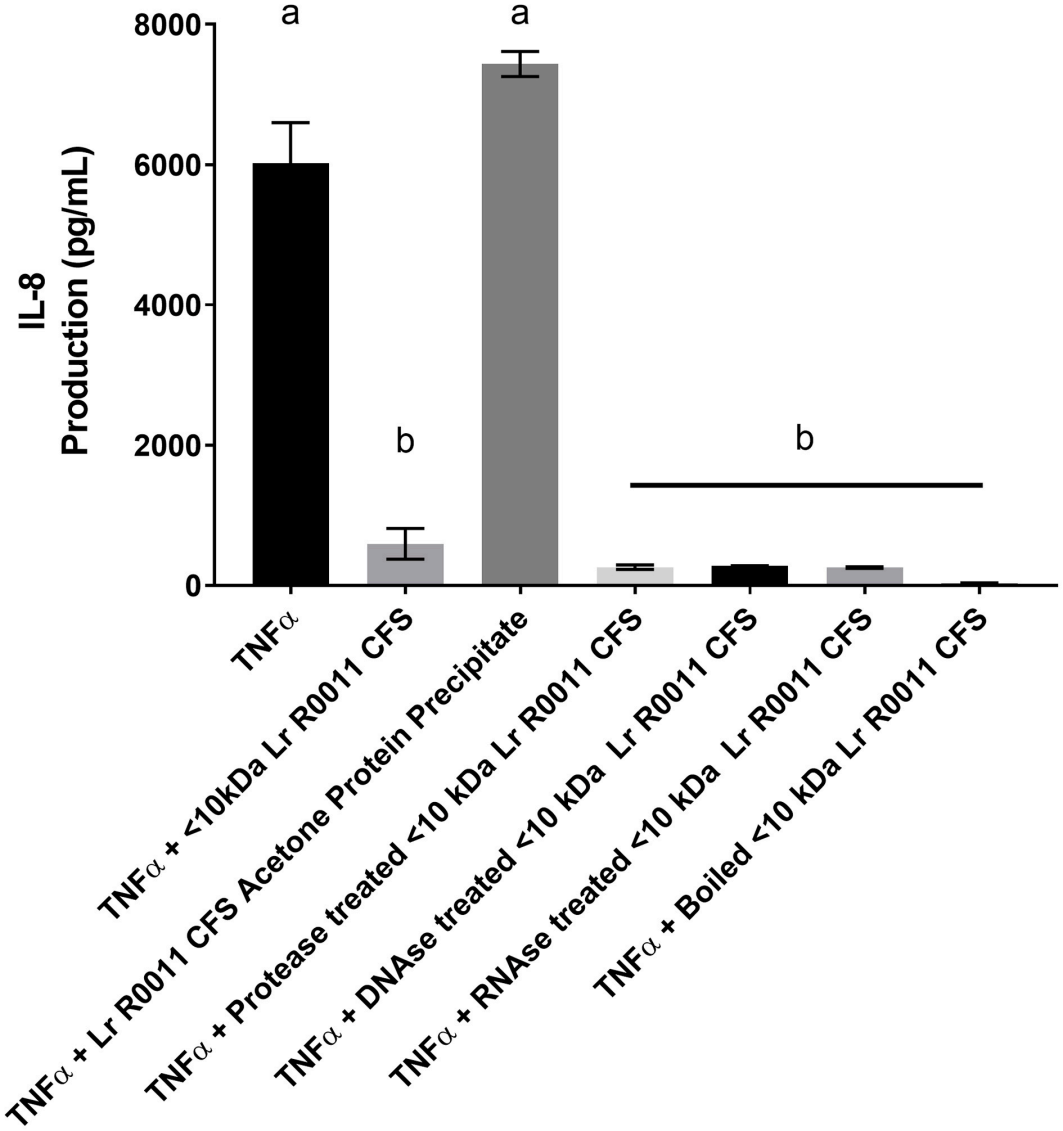
HT-29 IEC stimulated with TNFα and cultured with boiled Lr-CFS, an acetone precipitate of the Lr-CFS, or Lr-CFS treated with RNAse, DNAse or protease for 6 h (*n* = 3). Data shown are the mean IL-8 production ± SEM. Different letters between treatments denote significance (*P* < 0.05) as determined by one-way ANOVA and Tukey's *post-hoc* test.

**Figure 6:**
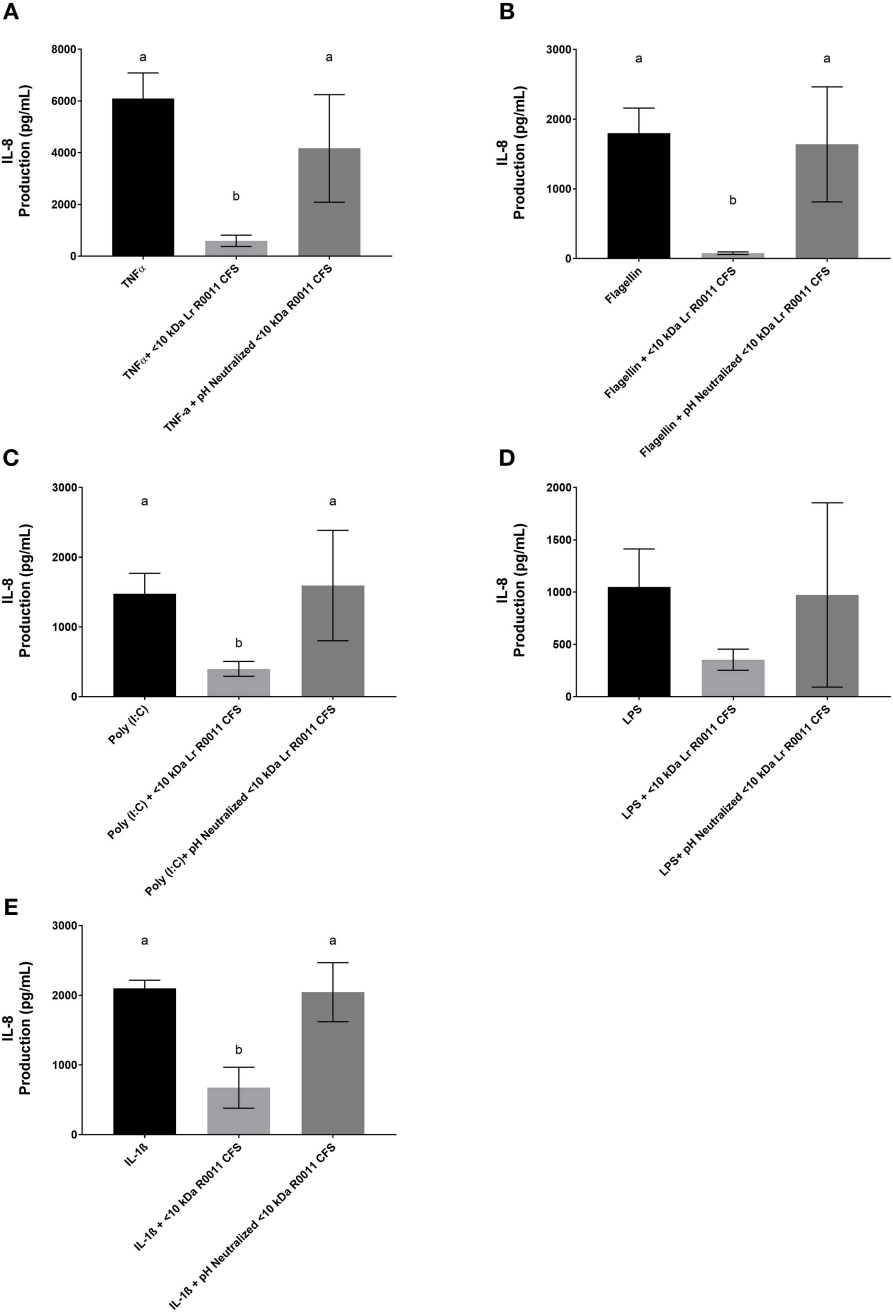
IL-8 production by HT-29 IEC challenged with PRR ligands, TNFα, or IL-1β and cultured with the <10 kDa fraction of the Lr-CFS or its pH-neutralized control (mean IL-8 production ± SEM; *n* = 6). **(A)** Lr-CFS and TNFα, **(B)** Lr-CFS and flagellin, **(C)** Lr-CFS and poly (I:C), **(D)** Lr-CFS and LPS, **(E)** Lr-CFS and IL-1β. Different letters denote significant differences between treatments (*P* < 0.05) as determined by one-way ANOVA and Tukey's *post-hoc* test.

**Figure 7:**
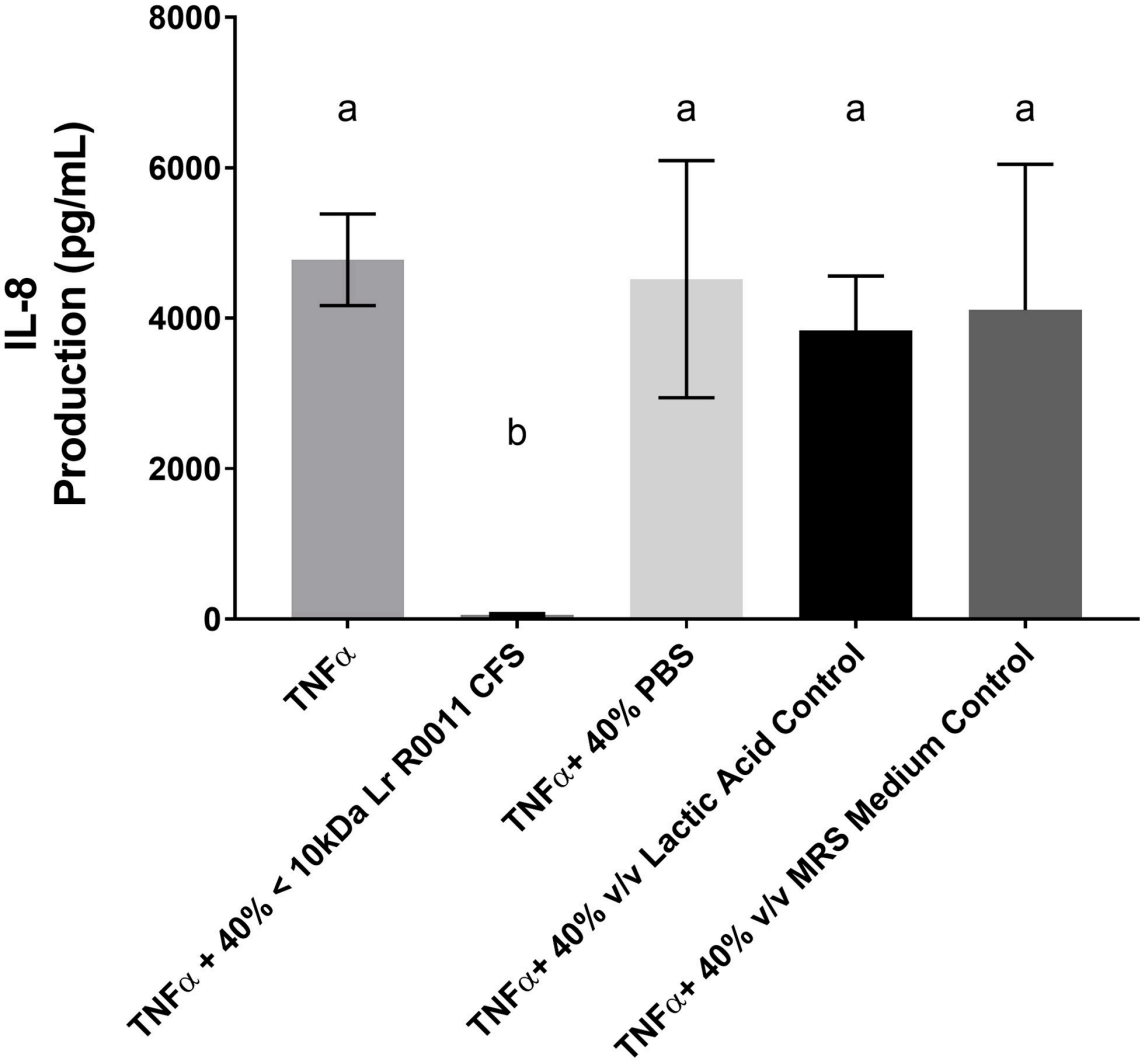
HT-29 IEC challenged with TNFα and cultured with 40% PBS controls, 40% MRS controls or L-lactic acid at a concentration equivalent to that present in the Lr-CFS (18.4 ± 1.7 mM; initial pH = 4.6; pH = 5.9 following 40% v/v dilution). Mean IL-8 production shown in pg/mL ± SEM (*n* = 4). Different letters denote significance between treatment groups as determined by one-way ANOVA and Tukey's *post-hoc* test.

To initiate characterization of the active components of the Lr-CFS, HT-29 IECs were challenged with TNFα and cultured with the <10 kDa fraction of the Lr-CFS which was heat-treated or treated with protease, RNase, DNase, or subjected to pH neutralization to physiological pH (pH = 7.4) using NaOH, and the effects on the ability of the CFS to decrease IL-8 production were measured. Lr-CFS which was boiled or treated with DNase, RNase, or an acidic protease retained the ability to significantly down-regulate IL-8 production (*P* < 0.05), while an acetone protein precipitate of the Lr-CFS did not (Figure 5). pH-neutralization of the Lr-CFS fraction reversed the bioactivity observed on HT-29 IECs challenged with TNFα, IL-1β, poly (I:C), or flagellin (Figure 6). To ensure the observed down-regulation of IL-8 production was not simply due to the lactobacilli culture medium, or a result of glucose and amino acid depletion in the RPMI-1640 medium by *L. rhamnosus* R0011, HT-29 IECs were also cultured with media controls containing PBS or MRS at 40% v/v. These controls had no effect on TNFα-induced IL-8 production by HT-29 IECs (Figure 7). L-lactic acid is the major fermentation product of *L. rhamnosus* R0011. To determine whether L-lactic acid was responsible for the effects of the Lr-CFS on chemokine production, L-lactic acid was quantified in the Lr-CFS, and was found to be present at a concentration of 18.4 ± 1.7 mM. This concentration of L-lactic acid was added to MRS/RPMI-1640 medium to serve as an L-lactic acid control (initial pH = 4.6; pH = 5.9 following 40% v/v dilution), and did not suppress TNFα-induced IL-8 production by HT-29 IEC (Figure 7).

To further explore the potential mechanism of action of the Lr-CFS, we examined the role of the ecto-5′-nucleotidase CD73 and the adenosine A_2_A receptor in mediating the effects on TNF-α-induced IL-8 production, as ATP was found in both Lr-CFS and Lh-CFS (41.7 vs. 9.2 nM, respectively). Adenosine generated from the catalytic conversion of ADP by CD73 helps shape immune responses to pro-inflammatory challenge (38) and the adenosine A_2_A receptor is involved in immunomodulation by certain lactobacilli (39). Although HT-29 IEC do express cell surface CD73, there were no significant differences in CD73 expression on HT-29 IEC cultured with the <10 kDa fraction of the Lr-CFS or any of the other treatments, and TNF-α did not induce increased CD73 expression (Supplemental Figure 1). Further, blocking of CD73 nucleotidase activity with adenosine 5′-(α,β-methylene) diphosphate [50 μM; (34)] did not reverse the down-regulatory activity of the Lr-CFS on TNF-α-induced IL-8 production. Blocking of the A_2_A adenosine receptor, with ZM241385, an A_2_A receptor-specific antagonist, also did not reverse the Lr-CFS immunomodulatory activity (Supplemental Figure 2). To determine whether the effects of the Lr-CFS were mediated through PPARγ, another route reported for immunomodulatory impact of certain lactobacilli (40), HT-29 IECs were cultured with GW9662, a selective PPARγ antagonist. The addition of this antagonist did not reverse the ability of the Lr-CFS to down-regulate TNFα-induced IL-8 production by HT-29 IEC (Supplemental Figure 2).

## Discussion

Several species and strains of LAB have been characterized for their ability to modulate host immune activity through direct interactions with the mucosal immune system. Varied strain-dependent host defense-associated activities have been reported for LAB, including stimulation of the immune system, modulation of host immune responses to pro-inflammatory challenges, and the ability to antagonize the attachment of known-gut pathogens to IECs (41). While mechanisms of action for certain strains, such as *L. rhamnosus* GG, have been determined (5, 18, 19, 42), they remain undefined for many others. The current study indicates that both *L. rhamnosus* R0011 and *L. helveticus* R0389 secrete bioactive molecules of <10 kDa in size that attenuate pro-inflammatory chemokine production from challenged human HT-29 IECs. Further, the *L. rhamnosus* R0011 CFS and its <10 kDa fraction decreased pro-inflammatory chemokine production from primary IEC-6 cells challenged with PRR ligands. These findings suggest that *L. rhamnosus* R0011 modulates IEC activity by secreting bioactive molecules that act to dampen signals delivered through key PRRs and cytokine receptors associated with pro-inflammatory activity.

These findings are in agreement with other studies suggesting that certain (LAB) secrete bioactive molecules with immunomodulatory activity. For example, cell-free supernatants from *L. kefir* IM002 have been shown to significantly inhibit IL-8 production from HT-29 IECs challenged with *Salmonella typhimurium* (43). *B. infantis* and *L. acidophilus* also secrete a bioactive molecule which down-regulated IL-8 and IL-6 production from IL-1β and TNF-α challenged primary human enterocytes through the modulation of genes involved in innate immunity (44). Recent findings support a role for interdomain communication via secreted molecules produced by other non-pathogenic bacteria, often referred to as the secretome (45, 46). For example, *E. coli* strain Nissle 1917 has been shown to produce a bioactive molecule that suppresses TNF-α-induced IL-8 production from human IECs in an NF-κB independent manner (47). Some pathogenic bacteria also secrete proteins and other molecules with bioactivity, suggesting this is an important route of microbe-host communication. For example, several species of Neisseria, including *Neisseria meningitidis*, secrete multiple proteins and other small molecules responsible for communication with the host (48, 49). Monosaccharide heptose-1,7-bisphosphate (HBP), a metabolic intermediate in LPS synthesis is released into the CFS of *N. meningitidis*, interacts with the novel PRR TRAF-interacting protein with forkhead-associated domain (TIFA) to activate NFκB and induce macrophage cytokine production (50). While these studies suggest varied roles for soluble mediators in bacteria-host interdomain communication, relatively few such mediators have been defined, and information about their impact on PRR or cytokine-induced signaling in innate immunity is currently limited.

Acidification of the cell culture medium to the pH of the Lr-CFS resulted in a degree of attenuation of IL-8 production in response to challenge with the pro-inflammatory cytokine IL-1β. This effect of acidification was not seen when HT-29 IECs were challenged with TNFα or with varied PRR ligands. Acidification of culture medium has been associated with diminished monocyte activity and TNFα secretion as a result of a decrease in the glycolytic flux in macrophages (51). Moreover, culture medium acidification results in reduction of IL-6 and IL-8 production from airway epithelial cells challenged with or without LPS (52). Overall this suggests a stimulus-dependent impact of acidification, which may contribute to the bioactivity of the Lr-CFS in HT-29 IECs challenged with IL-1β.

To initiate characterization of the bioactive molecule(s) present within the Lr-CFS, the <10 kDa fraction was subjected to treatment with DNase, RNase, protease, boiling, or pH alterations. Only neutralization of the pH of the CFS to physiological levels (pH = 7.4) with NaOH abrogated the down-regulation of TNF-α-induced IL-8 production from HT-29 IEC. This indicates that the bioactive molecule is pH sensitive and may provide insight into its molecular nature. A similar effect was also seen when the pH of the <10 kDa fraction of the CFS derived from *L. rhamnosus* GG was adjusted to physiological levels. When the pH of the <10 kDa fraction was increased from pH 4 to pH 7 using concentrated NaOH, the ability of the *L. rhamnosus* GG CFS to induce heat shock protein expression in IECs was lost (4). Furthermore, lipoteichoic acid (LTA) has been shown to be sensitive to sodium hydroxide treatment (53) LTA isolated from Enterococcus faecalis treated with sodium hydroxide or calcium hydroxide loses the ability to induce pro-inflammatory cytokine and nitric oxide production from RAW 264.7 cells (54). Interestingly, LTA isolated from some Gram-positive bacteria is smaller than 10 kDa in size (55). Although not characterized in this study, LTA from some (LAB) have been shown to modulate IL-8 production from HT-29 IECs in response to pro-inflammatory challenge. LTA isolated from *Lactobacillus* johnsonii La1 inhibits IL-8 production from TNF-α and LPS challenged HT-29 IEC, and deacylation of this LTA via ammonium hydroxide treatment reversed the anti-inflammatory effect (56). Whether an LTA component of the Lr-CFS or the Lh-CFS is involved in the impact on IL-8 production observed in the present study warrants further study.

To examine down-stream molecular targets of the secretome, HT-29 IEC were cultured with the selective PPARγ antagonist GW9662 to determine whether the bioactivity of the secretome is mediated through this nuclear receptor. It has been reported that some probiotic bacteria activate the PPARs, specifically PPARγ, through the local production of conjugated linoleic acid (CLA) (57). Orally administered probiotics have also been shown to protect against sepsis-induced liver and colonic damage in mice through the activation of PPARγ (58). However, the addition of the PPARγ antagonist GW9662 did not reverse the ability of the Lr-CFS to down-regulate TNF-α-induced IL-8 production from challenged HT-29 IEC, suggesting that the mechanism of action differs from other gut microbes shown to down-regulate NFκB activation through modulation of PPARγ receptor activity (57, 59, 60).

We have previously reported that *L. rhamnosus* R0011 induces an increase in intracellular cAMP production in HT-29 IEC, concurrent with down-regulation of LPS-induced IL-8 production (28). Activation of the adenosine A_2_A receptor has been shown to induce accumulation of intracellular cAMP, leading to inhibition of LPS-induced NFκB activity (61). Extracellular adenosine can act as an immunomodulatory molecule through activation of the A_2_A receptor (62). Adenosine can be generated from extracellular ATP and ADP through the catalytic activity of CD73, an ecto-5′-nucleotidase, and adenosine generated through this route helps direct host immune outcomes (38). As the adenosine A_2_A receptor is involved in the immunomodulatory activity of *Lactobacillus reuteri* (39) and commensal-derived ATP has been reported to have an impact on host immune function (63), we explored the possibility of CD73 and A_2_A receptor involvement in mediating the effects of the Lr-CFS on TNFα-induced IL-8 production. While we found that HT-29 IEC did express CD73, expression of this cell surface nucleotidase was unaffected by the Lr-CFS or by TNFα, and a CD73 antagonist did not reverse the immunomodulatory activity under the conditions examined in this study. Further, pharmacological blocking of the A_2_A adenosine receptor, the down-stream target of the liberated adenosine, also did not reverse the immunomodulatory activity of the Lr-CFS, suggesting that these receptors, although potentially important in certain microbe-host interactions, are not involved in the effects of the Lr-CFS on IEC.

In contrast to the suppression of poly (I:C)-induced IL-8 production from HT-29 IECs, Lr-CFS did not down-regulate poly (I:C)-induced CINC-1 production from IEC-6 IECs, and this was the only instance where the Lr-CFS did not down-regulate pro-inflammatory chemokine production from challenged IECs. This discrepancy may reflect differences between the species from which the two IEC lines were isolated or the location along the GI tract from which they originate, with potential differences in cytokine and TLR-induced signaling pathways (64, 65). TLR-3 signaling is unusual in that it can be mediated through a MyD88-independent pathway, while the other TLR stimuli used in this study act primarily through the MyD88-dependent pathway (66). TLR-3 activation by poly(I:C) challenge also results in differential activation of NFκB depending on the species of origin and cell lineage, with differences between human and murine poly(I:C)-induced NFκB activity and cytokine production suggesting differences in the signaling molecules involved in TLR-3-mediated responses (67). For example, exposure of HT-29 IEC to rotavirus induces activation of retinoic acid-inducible gene-1 (RIG-1) and the melanoma differentiation-associated gene-5 PRRs, leading to TLR-3-independent generation of pro-inflammatory biomarkers (68). Sequence analysis of stimulator of IFN genes (STING), a down-stream molecular target of RIG-1, in IEC-6 cells has revealed a 68% amino acid sequence similarity with human STING (69) suggesting that differences in poly(I:C)-induced signaling may underlie this difference in the observed immunomodulatory activity of the Lr-CFS between rodent and human IEC. Poly (I:C) can also induce HT-29 IEC to express 2′5′-oligoadenylate synthetase-like protein (OASL), another PRR involved in viral nucleic acid recognition (70), and it is possible that differences between HT-29 IEC and IEC-6 in expression of such alternate PRRs contributes to the differential impact of the Lr-CFS on TLR-3-mediated chemokine production. *In vivo* approaches aimed at delineating the differences in TLR-3 signaling in the intestinal epithelium along the GI tract will aid in furthering our understanding of these complex interactions between pathogens, commensal gut microbes, and the host in the gut mucosal environment.

When IECs were exposed to either the Lr-CFS or Lh-CFS without any innate immune stimulants, there were no alterations in the constitutive production of IL-8 or CINC-1 from HT-29 or IEC-6 IECs, respectively. This is in keeping with other studies indicating that certain (LAB) only exert their immunomodulatory activity when host cells are exposed to a pro-inflammatory stimulus. Transcriptomic analysis comparing HT-29 IEC cultured in the presence or absence of poly (I:C) indicates that a commercial probiotic mixture of *L. helveticus* R0052, *Bifidobacterium longum* subsp. infantis R0033, and *Bifidobacterium bifidum* R0071 only modulates genes involved in the inflammatory response when IEC are co-treated with both poly (I:C) and the probiotic mixture (70). Conversely, it has been shown that some lactobacilli can induce a pro-inflammatory response when in contact with IECs in the absence of any additional stimuli. For example, incubation of high concentrations of *L. rhamnosus* GG with Caco-2 IECs resulted in significantly higher levels of IL-8 production, without a requirement for TNF-α-induced signaling (71). The results of the present study suggest that the Lr-CFS and the Lh-CFS do not increase basal pro-inflammatory chemokine production, and that the observed immunomodulatory activity is only seen when IEC are exposed to pathogen or host-derived pro-inflammatory stimuli.

Collectively, our findings illustrate that *L. rhamnosus* R0011 and *L. helveticus* R0389 secrete bioactive molecules with immunomodulatory activity, and that these bioactive molecules can down-regulate IL-8 production by human HT-29 IECs induced by varied innate immune stimuli. The bioactive molecule(s) secreted by *L. rhamnosus* R0011 is <10 kDa in size and is sensitive to pH neutralization with sodium hydroxide. Moreover, these secreted factors also down-regulated CINC-1 production from challenged IEC-6 cells, with the exception of poly (I:C)-induced CINC-1 production. The CFS did not induce pro-inflammatory IL-8 or CINC-1 production in the absence of stimulus from PRR ligands or pro-inflammatory cytokines, suggesting that the observed immunomodulatory activity is only seen when IEC are in a pro-inflammatory environment. These findings reinforce and expand upon the current knowledge surrounding the ability of lactobacilli to communicate with host IEC, and may provide insight into potential mechanisms of action. However, key differences were observed between the immunomodulatory activity of these two probiotic strains depending on the cell-type and TLR ligand used suggesting potentially distinct mechanisms of action which require further investigation, as does the role of soluble mediators in communication between lactobacilli and host intestinal epithelial cells at the gut mucosal interface *in vivo*.

## Author contributions

Researchers in this group were involved in the original design of the project or contributed to experimental design throughout (MJ, JG-J, JS, and HJ). MJ carried out the experiments and analyzed the data. All authors were involved in preparation of the manuscript and approved the final version.

### Conflict of interest statement

The authors declare that the research was conducted in the absence of any commercial or financial relationships that could be construed as a potential conflict of interest.
