# Supplementary material for: Suppression of Intestinal Epithelial Cell Chemokine Production by Lactobacillus rhamnosus R0011 and Lactobacillus helveticus R0389 Is Mediated by Secreted Bioactive Molecules
Source: Front Immunol. 2018 Nov 14;9:2639. doi: 10.3389/fimmu.2018.02639 (PMC6262363; doi:10.3389/fimmu.2018.02639)
Supplement: Supplementary file 1 [file Data_Sheet_1.pdf]

frontiers

Supplementary Material

Suppression of intestinal epithelial cell chemokine production by *Lactobacillus rhamnosus* R0011 and *Lactobacillus helveticus* R0389 is mediated by secreted bioactive molecules

Michael P. Jeffrey[1], Janice L. Strap[1,2], Holly Jones Taggart[1,3], and Julia M. Green-Johnson*[1,2]

[1]Applied Bioscience Graduate Program and [2]Faculty of Science, [3]Faculty of Health Sciences, University of Ontario Institute of Technology, Oshawa, Ontario, Canada L1H 7K4.

*** Correspondence:**
Julia Green-Johnson, Faculty of Science, University of Ontario Institute of Technology, 2000 Simcoe Street North, Oshawa, Canada, L1H 7K4

Email Address: julia.green-johnson@uoit.ca

1.1 Supplementary Figures

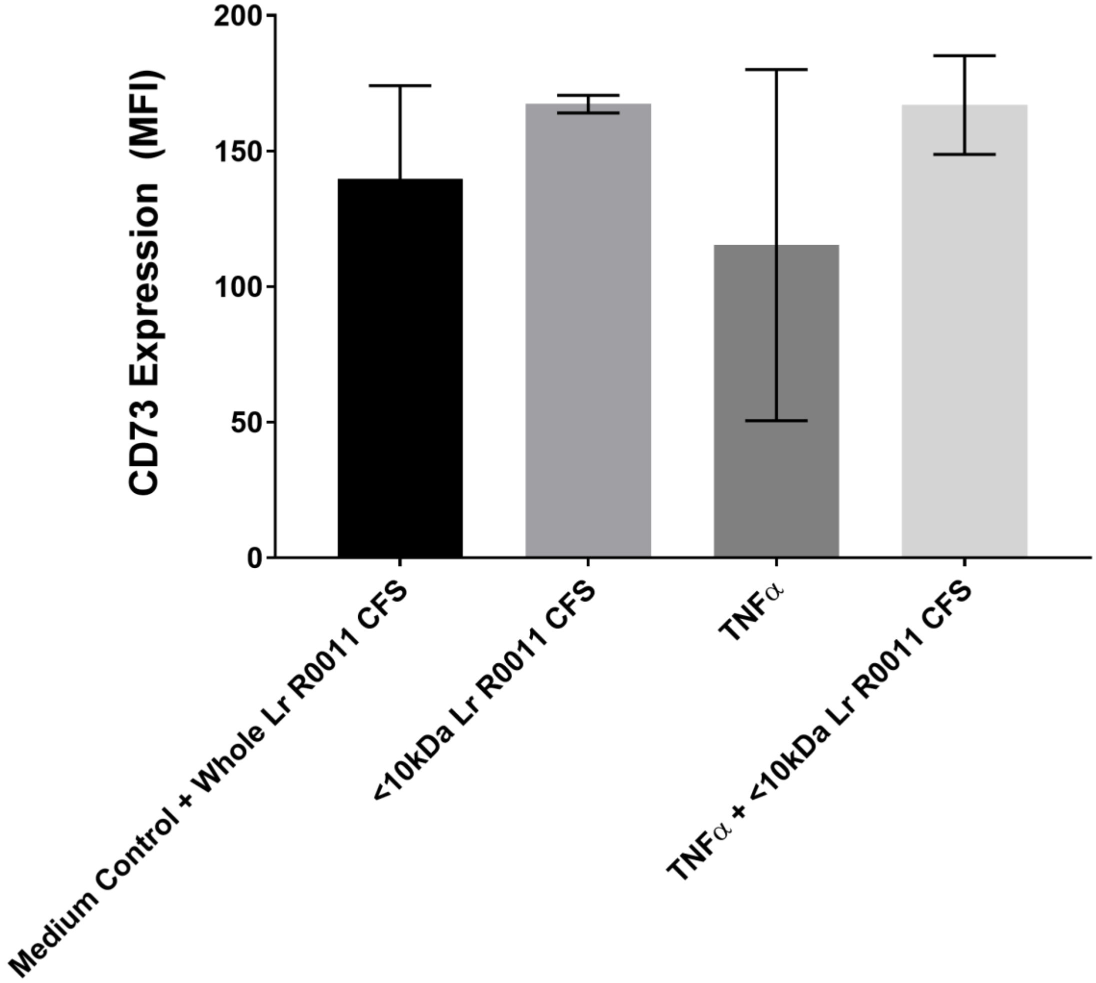

Supplemental Figure 1. HT-29 IEC express CD73 but expression levels are unaffected by TNFα or the < 10kDa fraction of the Lr-CFS. Data shown are the mean fluorescence intensity (MFI) ± SEM (n = 3). There were no significant differences between treatments as determined by the one-way ANOVA (P > 0.05).

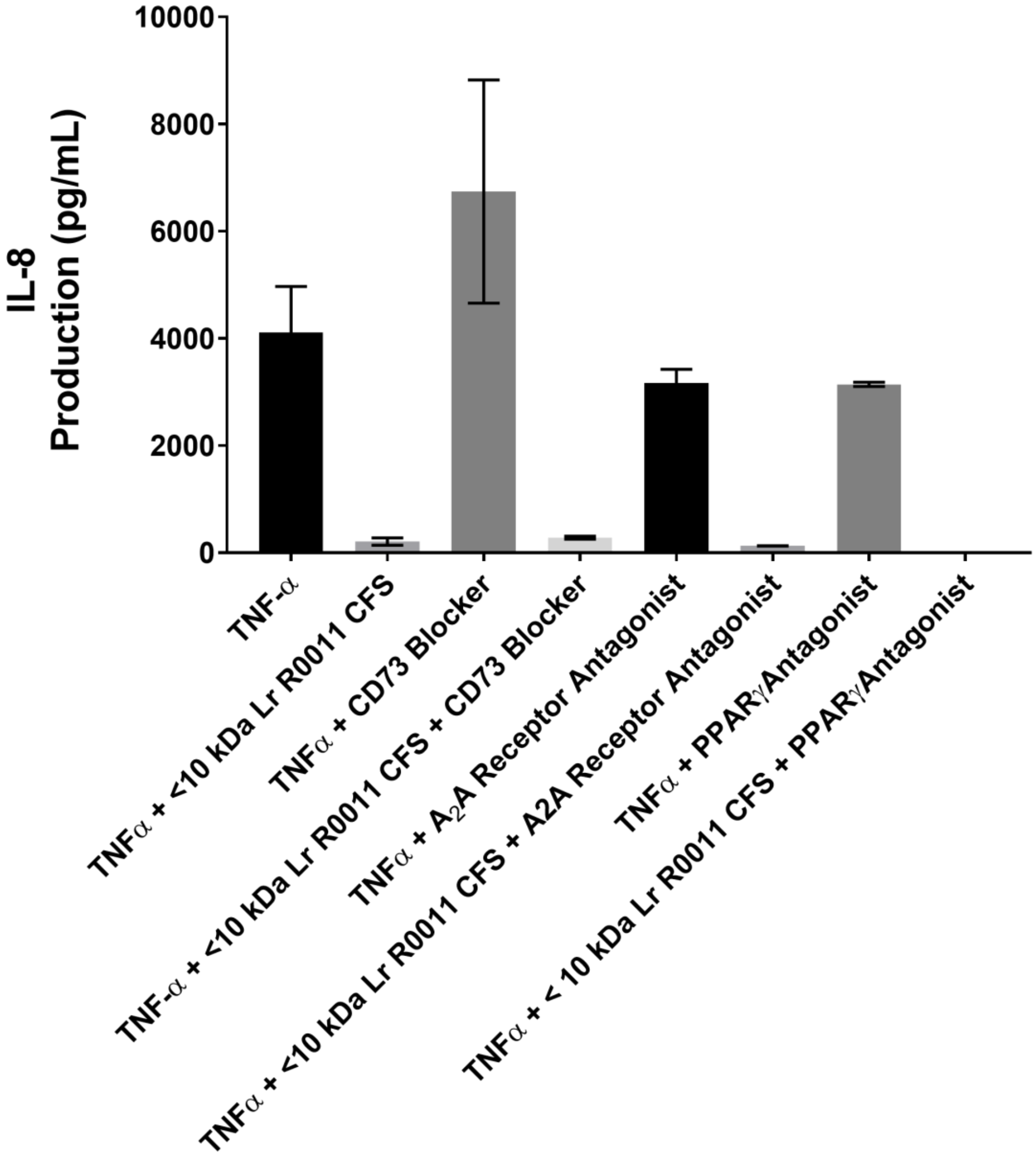

Supplemental Figure 2. TNFα-induced IL-8 production by HT-29 IEC cultured with the < 10kDa fraction of the Lr-CFS and a CD73 antagonist (adenosine 5'-(α,β-methylene) diphosphate) at 50 μM, an A2A receptor antagonist (ZM241385) at 500 nM, or a PPARγ antagonist (GW9662) at 5 μM for 6 hrs (mean IL-8 production ± SEM).
